# Utilizing Television Clips for Graduate Medical Education Anti-racist Curricula: An Acceptability Study

**DOI:** 10.7759/cureus.41526

**Published:** 2023-07-07

**Authors:** Beth L Hoffman, Jaime E Sidani, Charles R Jonassaint, Riley Wolynn, Anna K Donovan

**Affiliations:** 1 Behavioral and Community Health Sciences, University of Pittsburgh School of Public Health, Pittsburgh, USA; 2 Division of General Internal Medicine, University of Pittsburgh School of Medicine, Pittsburgh, USA

**Keywords:** medical television, mixed methods research, medical curricula, anti-racism, medical resident education

## Abstract

Introduction

Racism is a pervasive social problem that influences medicine, highlighting the need for interventions. One promising educational technique, referred to as edutainment, utilizes clips from television shows as an instructive strategy. The objective of this study was to examine the acceptability of edutainment around anti-racist curricula for residents.

Methods

We conducted a survey of underrepresented in medicine (URM) medical faculty to inform content for subsequent focus groups with medicine, psychiatry, and pediatrics residents. For the survey, URM faculty were randomly assigned to view four of eight clips and responded to close- and open-ended items. Focus group participants viewed selected clips and provided feedback. All study procedures occurred in 2020-2021. We calculated descriptive statistics for close-ended survey items and employed thematic analysis for open-ended items and focus group transcripts.

Results

Twelve URM faculty completed the survey. Feedback was uniformly positive so we included all eight clips in the resident focus groups. For each of the three participating specialties, we conducted two focus groups (2-11 participants each, total n=25) with participants viewing four of the eight clips. Analysis of focus group transcripts found that participants were receptive to the edutainment approach. Feedback as to the realism and acceptability of certain clips differed by specialty. Triangulation of survey and focus group results found differences in the acceptability of specific clips between residents and faculty.

Conclusion

Edutainment with medical television shows may be a promising avenue for anti-racist curricular content for residents. The educational methods described here are being incorporated into a multi-pronged, hospital system wide graduate medical education anti-racist curriculum.

## Introduction

Systemic racism continues to be a major problem plaguing every facet of American life; healthcare is no exception. Centuries of discrimination toward Black Americans have resulted in less access to appropriate education, funding, and encouragement, preventing them from having equal access to medical education [1]. Although Black individuals make up over 14% of the United States (U.S.) population, only about 7% of medical school matriculants identify as Black [2]. This lack of diversity has been linked to negative outcomes for minority patients, as racial concordance between patient and provider is associated with a greater patient satisfaction and greater likelihood of receiving preventive healthcare services [3,4].

While medical systems and training programs have focused heavily on the recruitment and retention of a racially and ethnically diverse workforce, there has been less emphasis on addressing workplace burdens experienced by trainees from racial and ethnic groups underrepresented in medicine (URM [5]. Specifically, URM trainees continue to face discrimination, social isolation, and different workplace expectations [5,6]. While accreditation agencies and institutions have called for graduate medical education (GME) anti-racist curricular content, there is a dearth of published interventions and evaluation for effective and scalable curricula that balance the need to address this potentially sensitive content area with psychological safety among trainees [5].

One promising educational technique, coined entertainment education or edutainment, involves the incorporation of educational content into entertainment content [7]. Sometimes edutainment is developed primarily for educational purposes, such as Sesame Street, although for adult audiences in the U.S. Edutainment more often involves including educational storylines in popular television programs or movies [7]. Prior research has found that viewing health storylines on both medical (e.g., Grey’s Anatomy) and non-medical television programs can influence viewers' health-related outcomes including perceptions of diseases and the healthcare workforce, knowledge, and behavioral interventions [8].

Edutainment can also refer to utilizing clips from television programs as part of health education interventions, both for lay populations and health sciences students [8,9]. Regarding the latter, research suggests that edutainment in the form of clips from popular medical television programs into medical curricula is feasible, efficacious, and more engaging for students than traditional lecture-type formats [10]. Prior work has used clips from medical programs such as ER and Grey’s Anatomy in conjunction with a brief lecture and small group work to improve trainee skills around inter-professional communication and having difficult conversations with patients [11,12]. Additionally, educators have previously used clips from House M.D. as part of an educational session for medical students to address anti-obesity bias, so we hypothesized that it would be a useful tool in teaching about race bias and anti-racism [13].

Prior to the development and implementation of such curricular content, there is a need to assess the acceptability of this approach with key partners in the clinical environment. Additionally, URM physician perspectives should be incorporated into anti-racism education. Therefore, to fill this gap, we conducted a study to assess the acceptability of this approach with URM faculty and residents from different specialties. This work was previously presented as a poster presentation at the 2022 Society of General Internal Medicine Annual Meeting on April 6, 2022, the 2022 University of Pittsburgh Department of Medicine Research Day on April 26, 2022, and the 2022 International Society for Research on Internet Interventions 11th Scientific Meeting on September 21, 2022. It was also presented as an oral presentation at the Rita M. Patel GME Leadership Conference on February 17, 2022.

## Materials and methods

URM faculty survey sample selection

Due to the COVID-19 pandemic increasing physician workforce demands and the paucity of URM medical faculty, it was determined that conducting focus groups with URM faculty would not be feasible. Therefore, we developed a survey to obtain URM faculty input on television clip selection for the resident focus groups and the use of edutainment for anti-racism GME curricula. Additionally, due to the paucity of URM medical faculty and increased URM medical faculty time demands for anti-racism initiatives in the aftermath of George Floyd’s murder, it was determined that convenience or random sampling would place an undo burden on URM medical faculty [6,14]. Therefore, we recruited URM medical faculty using purposive snowball sampling, whereby the authors emailed 10 medical faculties identifying as URM who were also involved in GME, inviting them to complete a brief electronic survey and share the invite with colleagues. We followed up with invited faculty to determine if they shared the invite with colleagues; in total, 15 URM faculty were invited to complete the survey. Inclusion criteria for completing the survey included identifying as URM, having a clinical terminal degree (e.g., MD, DO, PsyD), and having a faculty appointment in a School of Medicine in the U.S. The survey was closed for additional responses once thematic saturation was reached for responses to the open-ended items; all responses were recorded in Fall of 2020.

Participants received a $50 gift card upon completing the survey. Monetary or gift card incentives are standard practice to increase response rates to surveys, and compensating URM faculty for their time is one method for reducing the toll of the minority tax [6,15].

URM faculty survey development, procedures, and measures

We employed a three-step process to select television clips for the URM faculty survey. First, the lead author searched the Internet Movie Database (IMDB) and consulted with Hollywood, Health & Society to identify a set of 15 storylines on popular medical dramas (e.g., Grey’s Anatomy) addressing key concepts for anti-racist curricula identified in the literature, such as the minority tax [14,16,17]. The lead author then created a spreadsheet with a link to view each storyline, episode airdate, and a brief plot summary. Second, three other investigators with either lived experience as a URM faculty member or clinical educator individually viewed and provided feedback about the clips, with feedback compiled by the lead author.

Finally, the group discussed the compiled feedback and eliminated clips deemed unrealistic leaving a final set of eight clips with a plan to pair complementary clips (i.e., four pairs of two clips that addressed similar topics) (Table 1). The four topics represented in each pair of clips were (1) health disparities and stigmatizing language, (2) physician becoming jaded about the role of the healthcare system in addressing racism, (3) bias in clinical care, and (4) tokenism and the minority tax (Table 1). Using Qualtrics software, survey respondents were randomized to view either the first two pairs of clips (four clips total) or the last two pairs (Table 2).

**Table 1:** TV clips used, grouped by pair

Pair 1: Health Disparities and Stigmatizing Language		
Show and Episode	Description of Clip Content	Themes Addressed
ER 4.09 “Obstruction of Justice” (1997)	Young Black man presents with pain, and Carol says she can’t tell if he is a “junkie” or in a sickle cell crisis. The patient asks for meperidine, and Dr. Ross gives him hydromorphone but also coaches him through guided relaxation.	Stigmatizing language (toward patients); disease disparities
New Amsterdam 3.06 “Why Not Yesterday” (2020)	A series of people (both patients and providers) discuss the microaggressions they face daily.	Microaggressions; stigmatizing language (toward patients and providers); disease disparities
Pair 2: A Physician Becoming Jaded		
ER 3.17 “Tribes” (1997)	Dr. Carter, a surgical intern, treats a middle-aged Black woman who was sent over from a private hospital because she does not have insurance. The other hospital and attending physician both incorrectly assumed her pain was due to drug use or eating spicy food; she ends up having an MI.	Bias in clinical care; disease disparities; discussions with colleagues
ER 11.18 “Refusal of Care” (2005)	A Black female patient refuses a biopsy, and Dr. Pratt (Black doctor) explains to his White colleague why this patient is refusing the biopsy. Later, Dr. Pratt introduces the patient to a patient navigator.	Mistrust; discussions with colleagues; bias in clinical care
Pair 3: Bias in Clinical Care		
Grey’s Anatomy 17.04 “You’ll Never Walk Alone” (2020)	Asian-American patient previously diagnosed with appendicitis has right-sided diverticulitis, which is more common in Asian populations. A resident confronts Dr. Hunt about missing the diagnosis, and Dr. Hunt and Dr. Bailey discuss equity versus equality.	Disease disparities; bias in clinical care; discussions with colleagues
The Resident 2.20 “If Not Now, When?” (2019)	A White OBGYN ignores a Black postpartum woman’s medical decline and she dies. This was inspired by a true story and several physicians question if the lead OBGYN’s medical decisions related to the race of the patient.	Disease disparities; bias in clinical care; discussions with colleagues
Pair 4: Tokenism and the Minority Tax		
Scrubs 1.08 “My Fifteen Minutes” (2001)	Dr. Kelso uses images of Turk to push Sacred Heart as being an “inclusive” hospital. Turk pushes back and discusses feeling like the token Black doctor.	Tokenism; White colleagues not understanding Black colleagues' experience
ER 7.12 “Surrender” (2001)	Dr. Romano appoints Dr. Benton to be the new director of diversity as he is the only Black physician available.	Tokenism; White colleagues not understanding Black colleagues' experience

**Table 2:** Faculty survey items

Survey A: Clips 1-4		
Number	Question Text	
1.	The clip is from the television show ER and first aired in 1997. In the clip, a young Black man presents with pain, and Carol (a nurse) says she cannot tell if he is a "junkie" or in a sickle cell crisis. The patient asks for meperidine, and Dr. Ross gives him hydromorphone but also coaches him through guided relaxation.	
2.	Please rate the extent to which you agree or disagree with the following statements about the clip (scale from strongly disagree (1) to strongly agree (5)). 1. This clip will spark important discussions about race; 2. This clip portrays issues that are important for residents to discuss related to race; 3. I support using this clip as part of an educational session for residents on race; 4. The portrayal of issues around race in this clip was realistic based on my clinical experience	
3.	What are your overall thoughts about using the clip as part of a curricular intervention for resident physicians? If the clip was used, what are the key talking points for educators to bring up?	
4.	Any other comments or reflections after viewing this clip?	
5.	The clip is from the television show New Amsterdam and first aired in 2021. In the clip, a series of characters (both patients and providers) discuss the microaggressions they face.	
6.	Please rate the extent to which you agree or disagree with the following statements about the clip (scale from strongly disagree (1) to strongly agree (5)). 1. This clip will spark important discussions about race; 2. This clip portrays issues that are important for residents to discuss related to race; 3. I support using this clip as part of an educational session for residents on race; 4. The portrayal of issues around race in this clip was realistic based on my clinical experience.	
7.	What are your overall thoughts about using the clip as part of a curricular intervention for resident physicians? If the clip was used, what are the key talking points for educators to bring up?	
8.	Any other comments or reflections after viewing this clip?	
9.	We are thinking about pairing this clip with the previous clip to highlight how the patients with sickle cell anemia in both clips - one from 1997 and one from 2021 - experience dismissal of their pain. This could be used to invite discussion about what has/has not changed in medicine over the course of the last 25 years. What are your thoughts about pairing these clips together and these discussion points?	
10.	The clip is from the television show ER and first aired in 1997. In the clip, Dr. Carter, a surgical intern, treats a middle-aged Black woman who was sent over from a private hospital because she does not have insurance. The other hospital and Dr. Greene (attending physician) both assumed her pain was due to drug use or eating spicy food. Dr. Carter's workup reveals that she has an MI.	
11.	Please rate the extent to which you agree or disagree with the following statements about the clip (scale from strongly disagree (1) to strongly agree (5)). 1. This clip will spark important discussions about race; 2. This clip portrays issues that are important for residents to discuss related to race; 3. I support using this clip as part of an educational session for residents on race; 4. The portrayal of issues around race in this clip was realistic based on my clinical experience.	
12.	What are your overall thoughts about using the clip as part of a curricular intervention for resident physicians? If the clip was used, what are the key talking points for educators to bring up?	
13.	Any other comments or reflections after viewing this clip?	
14.	The clip is from the television show ER and first aired in 2005. In the clip, a Black female patient refuses a biopsy, and Dr. Pratt explains to his White colleague why she holds some of the beliefs she does. Dr. Carter is now the attending physician, and he tells Dr. Pratt to discharge her. However, Dr. Pratt introduces the patient to a patient navigator to facilitate her care.	
15.	Please rate the extent to which you agree or disagree with the following statements about the clip (scale from strongly disagree (1) to strongly agree (5)). 1. This clip will spark important discussions about race; 2. This clip portrays issues that are important for residents to discuss related to race; 3. I support using this clip as part of an educational session for residents on race; 4. The portrayal of issues around race in this clip was realistic based on my clinical experience.	
16.	What are your overall thoughts about using the clip as part of a curricular intervention for resident physicians? If the clip was used, what are the key talking points for educators to bring up?	
17.	Any other comments or reflections after viewing this clip?	
18.	We are thinking about pairing this clip with the previous clip to highlight how sometimes doctors "do the right thing" and sometimes they do not. This could be used to invite discussion about how people sometimes become "hardened" the longer they are in practice, and the importance of continuing to examine one's biases. What are your thoughts about pairing these clips together and these discussion points?	
Survey B: Clips 5-8		
1.		The clip is from the television show Grey's Anatomy and first aired in 2020. In the clip, an Asian-American surgical resident experiences a microaggression from a patient. Then, an Asian-American patient previously diagnosed with appendicitis has right-sided diverticulitis, which is more common in Asian populations. A resident confronts Dr. Hunt about missing the diagnosis, and Dr. Hunt and Dr. Bailey discuss equity versus equality.
2.		Please rate the extent to which you agree or disagree with the following statements about the clip (scale from strongly disagree (1) to strongly agree (5)). 1. This clip will spark important discussions about race; 2. This clip portrays issues that are important for residents to discuss related to race; 3. I support using this clip as part of an educational session for residents on race; 4. The portrayal of issues around race in this clip was realistic based on my clinical experience.
3.		What are your overall thoughts about using the clip as part of a curricular intervention for resident physicians? If the clip was used, what are the key talking points for educators to bring up?
4.		Any other comments or reflections after viewing this clip?
5.		The clip is from the television show The Resident and first aired in 2019. In the clip, a white OBGYN and white nurse both ignore a Black postpartum woman's medical decline despite a resident advocating for her. The patient dies and several physicians confront the OBGYN about his biases. This was inspired by a true story.
6.		Please rate the extent to which you agree or disagree with the following statements about the clip (scale from strongly disagree (1) to strongly agree (5)). 1. This clip will spark important discussions about race; 2. This clip portrays issues that are important for residents to discuss related to race; 3. I support using this clip as part of an educational session for residents on race; 4. The portrayal of issues around race in this clip was realistic based on my clinical experience.
7.		What are your overall thoughts about using the clip as part of a curricular intervention for resident physicians? If the clip was used, what are the key talking points for educators to bring up?
8.		Any other comments or reflections after viewing this clip?
9.		We are thinking about pairing this clip with the previous clip to highlight bias in clinical care and discuss equity versus equality. What are your thoughts about pairing these clips together and these discussion points?
10.		The following clip is from the television show Scrubs and first aired in 2001. In the clip, Dr. Kelso uses images of Dr. Turk to push Sacred Heart Hospital as being an inclusive hospital. Dr. Turk pushes back and discusses feeling like the token Black doctor.
11.		Please rate the extent to which you agree or disagree with the following statements about the clip above (scale from strongly disagree (1) to strongly agree (5)). 1. This clip will spark important discussions about race; 2. This clip portrays issues that are important for residents to discuss related to race; 3. I support using this clip as part of an educational session for residents on race; 4. The portrayal of issues around race in this clip was realistic based on my clinical experience.
12.		What are your overall thoughts about using the clip as part of a curricular intervention for resident physicians? If the clip was used, what are the key talking points for educators to bring up?
13.		Any other comments or reflections after viewing this clip?
14.		The clip is from the television show ER and first aired in 2001. In the clip, Dr. Romano appoints Dr. Benton to be the new director of diversity as he is the only Black physician available.
15.		Please rate the extent to which you agree or disagree with the following statements about the clip (scale from strongly disagree (1) to strongly agree (5)). 1. This clip will spark important discussions about race; 2. This clip portrays issues that are important for residents to discuss related to race; 3. I support using this clip as part of an educational session for residents on race; 4. The portrayal of issues around race in this clip was realistic based on my clinical experience.
16.		What are your overall thoughts about using the clip as part of a curricular intervention for resident physicians? If the clip was used, what are the key talking points for educators to bring up?
17.		Any other comments or reflections after viewing this clip?
18.		We are thinking about pairing this clip with the previous clip to highlight the issue of tokenism and the extra burden often placed on Black physicians by leadership. What are your thoughts about pairing these clips together and these discussion points?

For each clip, respondents were presented with a brief synopsis and a link to view the clip in their browser. To assess acceptability, we asked respondents to rate their agreement with four items on a Likert scale of one (strongly disagree) to five (strongly agree) and to complete two open-ended questions about each clip. We also asked one open-ended item after each pair about using the clips together. The full survey instrument is available in Table 2.

Resident focus group sample selection

We coordinated with internal medicine (IM), psychiatry, and pediatric residency leadership at our institution to send recruitment emails via residency program listservs at our institution. Focus groups were conducted during normal residency didactic times and approved by each program. All residents in each of the three programs were eligible to participate in the focus groups.

Resident focus group procedures

We conducted two small focus group discussions with residents in each specialty (six groups total) from October to December 2021. No personnel affiliated with residency leadership attended the discussions. Focus groups were facilitated by the first author with one other investigator present to take notes and lasted approximately one hour. Focus groups were conducted in person for IM and pediatrics and via Zoom videoconferencing software for psychiatry. Audio was recorded for transcription.

Focus group participants independently completed a brief socio-demographic survey and then viewed two pairs of clips (four total) as a group, with one focus group per specialty viewing the first two pairs and the other viewing the last two. Focus group questions were developed in consultation with the University of Pittsburgh Qualitative, Evaluation, and Stakeholder Engagement Research Service.

Analysis

For the survey, we calculated descriptive statistics for close-ended items using Stata 17 (StataCorp. 2021. Stata Statistical Software: Release 17. College Station, TX: StataCorp LLC) and used thematic analysis by two independently working researchers for the open-ended items [18]. Two researchers independently examined the responses to identify themes related to acceptability and key talking points for educators. The researchers then met to discuss emergent themes and representative quotations [18].

We transcribed focus group recordings using Otter.ai and entered the transcripts and notes into NVivo software for deductive content analysis [18]. Two researchers independently coded the first focus group transcript using a draft codebook informed by the survey results, modifying the codebook based on direct analysis. Researchers met to codify a final codebook, which was used to independently double-code all six transcripts. Following coding, the researchers met to discuss their findings, adjudicate differences, and identify exemplar quotes. This study was deemed exempt by the University of Pittsburgh Institutional Review Board. All study procedures occurred in 2020-2021.

## Results

URM faculty survey

**Figure 1:**
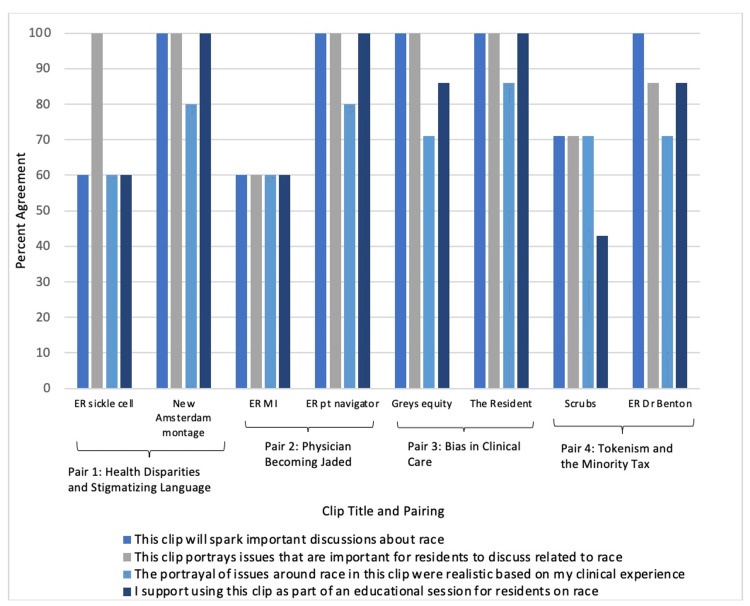
URM faculty survey respondents' percent agreement with each statement (n=5 for the first four clip titles and n=7 for the last four clip titles)

Twelve URM faculty completed the survey, with five respondents viewing the first four clips and seven viewing the second four. Percent agreement for each statement was >80% for most items (Figure 1); the only clip for which less than half of respondents agreed with the statement “I support using this clip as part of an educational session for residents on race” was the clip from Scrubs (Table 1). For the open-ended items, two prominent themes were that a clip (1) portrayed important issues about racism in medicine and (2) would spark discussions among trainees (Table 3). Several respondents noted clips could evoke emotions among trainees (e.g., wow, super powerful, and there would be lots of emotions to this one) and suggested trigger warnings for some clips. Responses to the open-ended items about pairing clips found respondents were supportive of this approach (Table 3).

**Table 3:** Coding categories, descriptions, and exemplary quotations

Name	Description	Example Quotations From Faculty Survey	Example Quotations From Resident Focus Groups
Use in Education	Supports (or not) using clip as part of GME anti-racist curricula	1. Excellent! 2. The clip could be useful as long as it's examined both from the patient's perspective and the physician's perspective.	1. I really like this format . . . to see dynamics of like residents and attendings and nurses and admin and, and kind of be able to watch the whole situation and then talk about it is really helpful. 2. [The clips] are definitely really good. I think adding one where they actually theoretically have more time to interact with a patient could also be helpful.
Discussions About Race	Mentions clip will spark important discussions about race dialogue among participants suggests clip will spark important discussions about race	1. This I think will spark dialogue about the issues of Black maternal death. 2. This is a nice clip as a springboard to discuss structural bias in treatment protocols and to have a discussion around equity.	1. It really demonstrates unconscious bias, especially just in the way that the old White surgeon is kind of thinking, in the way that people are not realizing that they're delivering non-equitable care. 2. We have the same thing [as in the clip] here. There is a hall full of old White men who were big names in the hospital that we pass by when we go to the lecture hall.
Portrays Important Issues	Clip portrays issues important for residents to discuss related to race and anti-racism	1. It’s pretty realistic and common encounter. 2. This clip was excellent. Just treating patients "equally" is not enough and can be detrimental. Use your privilege to make systematic changes.	1. I think Bailey has a great point at the end, which is everyone has biases; it's almost impossible to get rid of them entirely. 2. I liked when she said, it's not my job to dismantle systemic racism. I'm suffering from it. That's very important.
Realism	Clip is/is not realistic based on clinical experience	1. It's pretty intense - VERY REALISTIC. Would need a trigger warning. 2. This one didn’t seem as realistic, overly dramatized but certainly brings up feelings we have all experienced.	1. Yeah, it was a little, a little, Hollywood. 2. I thought it was a really realistic portrayal in terms of the patient and her concerns and also the dichotomy between the two physicians and their attitudes spanning the spectrum of what we perceive as right.
Key Talking Points	If clip was used, what are key talking points for educators to bring up?	1. Maybe you would want to talk about how to communicate the urgency and when to superiors. 2. Educators should focus the discussion on the broader take-home points and "broad" practical strategies to avoid microaggressions	1. This clip helps to show that while we use descriptors to say that this is the patient and this is their past medical history, it's really important to be more thoughtful about the words that we use. 2. That would be an opportune time to kind of say, “Okay, how would you address this with the nurse or, you know, would you address this?”
Differences in Specialties	Mentions differences between specialties or specific specialty	None	1. We are in the peds world, right? I think we move at a slower pace and we do take time to address a lot of these things. 2. That was a like, shockingly more in touch with society clip than I'd expect from Grey's Anatomy. A lot of that we talk about today, like there was a big movement last year about how dermatology was very White centric.
Pairing	Thoughts about pairing clips together or choosing one clip over another	1. Pairing this with a positive example could be effective and avoid overwhelming trainees. 2. [Pairing] could work, but I wasn't a fan of the first clip overall.	1. I'm not sure what the point of showing these clips together. 2. I feel like it works to have both of those clips, partly because, like we already pointed out, a lot of these things haven't changed in 20 years.

Resident focus groups

Focus group sizes ranged from two to nine participants (median=4), with a total of 25 participants across the three specialties. The focus groups where participants viewed the first two pairs of clips included two, four, and four participants (total=10), and the focus groups where participants viewed the second two pairs included two, four, and nine participants (total=15). The majority (56%, n=14) were either PGY-1 or PGY-2, 26-30 years of age (64%, n=16), and female (64%, n=16). Approximately half (48%, n=12) identified as White and 12% (n=3) as Black or African-American.

Overall, participants supported using the clips as part of training (Table 3). One theme that emerged across both the Discussions About Race and Portrays Important Issues coding categories was that the clips were helpful for sparking discussion (e.g., “having [a character] to talk about … about instead of a situation where everybody knows that you’re talking about this one attending I just think opens the door for more conversation”) (Table 3). 

Regarding realism, some participants thought certain clips were overly dramatic (e.g., “I felt like he was practicing … an entertaining spin on paternalistic medicine”), while others were realistic (e.g., “Almost every single thing [in the clip] I’ve seen or heard about”) (Table 3). Of note for pair 3 (Bias in Clinical Care), perceptions of realism differed by specialty, with IM residents thinking the clip from The Resident was realistic while residents from pediatrics and psychiatry thought that clip was unrealistic, and vice versa (i.e., IM residents thought the clip from Grey’s Anatomy was unrealistic while residents from pediatrics and psychiatry thought that clip was realistic; Table 1). Overall, participants indicated support for the Use in Education of these clips, noting they could be especially useful to provoke further dialogue. Several participants specifically mentioned support for the clip from Scrubs (e.g., “I just love Scrubs”). Focus group participants across specialties noted similar key take-home points for each clip but overall did not generally support pairing of the clips.

## Discussion

Results from this mixed-methods study support the use of edutainment for GME anti-racist curricular content. Resident participants felt the edutainment approach was impactful, largely realistic, and created psychological safety for the discussion of emotionally sensitive concepts. Interestingly, we found some differences between faculty and resident participants. The most notable difference between faculty and resident participants was related to the clip from Scrubs, with faculty less supportive of using this clip compared to residents. This clip was the only one in our set from a comedy program, and it may be that faculty were unsure if the comedic approach to a serious topic would be appropriate in the educational context. Faculty participants may have been less familiar with the characters from Scrubs, and therefore less likely to identify the humorous banter between characters on the show. Given the popularity of Scrubs among residents and health sciences students, future research could examine support for edutainment with clips from comedies compared to dramas [19].

We also found differences in perceptions of realism among residents of different specialties, supporting the importance of tailored approaches to curricular design. The varied feedback suggests that further curriculum development could be enhanced by co-creating content with individuals from the specific target groups of faculty and residents. Including learners in the design and conduction of educational initiatives has been recommended by the International Association for Health Professions Education (AMEE) as a technique for increasing learner motivation [20].

Given increasing societal discussions about race bias and race disparities in healthcare as well as the increasing call from medical education leaders to address these disparities in training, it is essential to identify effective ways to teach these concepts [21]. Specifically, prior reviews have demonstrated that many educational interventions focused on implicit bias employ a two-pronged strategy that includes first alerting students and trainees to the presence of their implicit biases and next educating them on methods to both reduce activation of these biases and reduce the influence of these biases on their clinical judgment and behavior [22]. Subsequent interventions have used multimodal educational approaches, including communication skills training utilizing implicit association tests, lectures, small groups, reading assignments, and simulated communication videos [23,24]. Although straightforward, this type of educational delivery can at times feel confrontational and cause learners to be defensive [25,26].

Edutainment, in contrast, aims to first engage participants with relatable scenarios before highlighting biases that may be relevant to the learner and their team. However, to the best of our knowledge clinician educators have not yet employed an edutainment approach for race bias education among resident trainees; furthermore, we did not identify any similar curricula for medical students or faculty learners. This edutainment approach may be one aspect of a successful and comprehensive multi-pronged approach to promoting racial justice and reducing health inequity in GME. Results from the current study, specifically resident focus group participants stating the clips were a helpful jumping-off point for discussion, suggest that an edutainment approach may “disarm learner defensiveness” and promote the psychological safety called for by Blanchard et al. (2022) to discuss these challenging topics [17]. Additionally, through mechanisms such as character identification and narrative transportation, edutainment may reduce resistance to discussing potentially uncomfortable topics, which could improve knowledge retention and foster more meaningful discussion compared to more traditional pedagogical approaches [27]. 

Moreover, given that medical professionals on television are more racially diverse than in real-life, the edutainment approach may be particularly impactful in reducing implicit bias among residents by exposing them to diverse characters and storylines that challenge preconceived notions and stereotypes [28]. This exposure could promote greater empathy and understanding, playing a crucial role in cultivating culturally sensitive healthcare providers who can deliver equitable care to diverse patient populations [29,30].

Despite the numerous possible advantages to an edutainment approach in anti-racism curricula, educators undertaking this work must also employ a framework of cultural competence to navigate potentially challenging and emotionally charged discussions that may follow edutainment clips that begin such discussions. With diverse groups of participants, educators facilitating discussion need to understand various perspectives, defuse conflict, and have a trauma-informed approach to racist and microaggressive comments. Depending on the pre-existing skills and interests of the educators, it is expected such a curriculum would require faculty development for group facilitators. Furthermore, the authors recommend that educators for these sessions maintain confidentiality and emphasize that they are not evaluating participants for competency or promotion in a summative way, as this may undermine psychological safety for group discussion. Finally, edutainment curricula are limited by the television clips that are available in terms of content/topic, emotional depth, and diversity of actors portraying characters; the extent of the clips has not yet limited our work in this area but is a conceivable limitation for future work.

Our results should be interpreted with the following limitations. First, while the use of snowball sampling for recruitment was deemed most feasible given the COVID-19 pandemic and increased URM physician workforce demands, it limited our sample size and limits the generalizability of our survey findings. Moreover, although we had no reason to suspect receipt of survey responses from individuals who did not meet our inclusion criteria, we did not conduct formal eligibility screening. Future work could use convenience or random sampling to both increase the number of respondents and the generalizability of our findings to the broader URM medical faculty workforce. Similarly, several of our focus groups were small and our focus groups included residents from only three specialties. We attempted to involve six large specialties but were unsuccessful in engaging three of them during the COVID-19 era with its clinical demands and workforce challenges. Larger system-wide efforts have been more successful in engaging other programs and specialties in a multimodal GME effort incorporating our approach. While thematic saturation was achieved with the open-ended survey responses and focus groups, future research could expand this work to additional specialties. Additionally, participants who opted into the study may have been more likely to watch medical television programs and more engaged in race bias mitigation efforts than those who did not. Future research could employ a randomized design whereby participants watch these clips or another instructional tool, to compare the two modalities. A mandatory requirement for participation may also reduce the effects of selection bias among participants.

## Conclusions

Our findings suggest edutainment may be a promising avenue for GME anti-racist curricular content, and specifically that edutainment may offer a way to promote psychological safety when discussing this potentially sensitive content area. Our study also highlights the value of formative research in developing such curricula, with differences observed between faculty and resident participants and residents of different specialties supporting the use of gathering input from a diverse group of participants and tailored approaches to curricular design. Future work could use these findings to pilot anti-racist faculty development sessions and educational workshops for residents that incorporate the clips utilized in this study; our team is currently conducting such work as part of a multi-pronged, hospital system wide GME anti-racist curriculum. Future research could also conduct focus groups with trainees from other specialties or identify and obtain feedback on different clips or media content (e.g., social media videos).
